# Self-Care in Heart Failure Inpatients: What Is the Role of Gender and Pathophysiological Characteristics? A Cross-Sectional Multicentre Study

**DOI:** 10.3390/healthcare9040434

**Published:** 2021-04-08

**Authors:** Bruno Delgado, Ivo Lopes, Tânia Mendes, Patrícia Lopes, Luís Sousa, Fidel López-Espuela, Leonel Preto, Eugénia Mendes, Bárbara Gomes, André Novo

**Affiliations:** 1 Centro Hospitalar Universitário do Porto—Hospital de Santo António, Instituto de Ciências Biomédicas Abel Salazar—Universidade do Porto, 4050-313 Porto, Portugal; bruno.m.delgado@gmail.com; 2 Centro Hospitalar do Porto—Hospital de Santo António, 4099-001 Porto, Portugal; enf.ivo.lopes@gmail.com; 3 Instituto Português de Oncologia, 4200-072 Porto, Portugal; tania_rbsm@hotmail.com; 4 Centro Hospitalar de Vila Nova de Gaia/Espinho, 4434-502 Vila Nova de Gaia, Portugal; enfpatricialopes@gmail.com; 5 Comprehensive Health Research Centre (CHRC), Department of Nursing, University of Évora, 7000-812 Évora, Portugal; luismmsousa@gmail.com; 6 Metabolic Bone Diseases Research Group, Nursing Department, Nursing and Occupational Therapy College, University of Extremadura, 06071 Badajoz, Spain; 7 The Health Sciences Research Unit: Nursing (UICISA: E), Instituto Politécnico de Bragança, 5300-253 Bragança, Portugal; leonelpreto@ipb.pt; 8 Departamento de Enfermagem, Instituto Politécnico de Bragança, 5300-253 Bragança, Portugal; maria.mendes@ipb.pt; 9 Escola Superior de Enfermagem do Porto, 4200-072 Porto, Portugal; bgomes@esenf.pt; 10 Center for Health Technology and Services Research (CINTESIS) NursID, Instituto Politécnico de Bragança, 5300-253 Bragança, Portugal

**Keywords:** heart failure, physical activity, gender, nursing care, self-care behaviour

## Abstract

Heart failure is often characterised by low exercise capacity and a great impairment of performance in the activities of daily living. The correct management of the disease can prevent the worsening of symptoms and promote a better quality of life. The aims of this study are to understand the relationship of gender and pathophysiological characteristics with self-care behaviour and to evaluate the self-care behaviour in a sample of Portuguese heart failure inpatients, using the Self-Care of Heart Failure Index (SCHFI). A cross-sectional multicentre study enrolling 225 heart failure inpatients from eight hospitals from Portugal was performed. At admission, each patient’s functional capacity was evaluated as well as their self-care behaviour, using the SCHFI Portuguese v6.2. A comparison between self-care behaviour with gender was performed. The patients’ mean age was 68.4 ± 10.7 years old, 68% were male and 82.3% had reduced ejection fraction. A mean value of 47.9, 35.6 and 38.8 points was found in the SCHFI score of the sections self-care maintenance, self-care management and self-care confidence, respectively. Heart failure inpatients present inadequate levels of self-care behaviour. The results do not suggest a relationship between gender and pathophysiological characteristics with self-care behaviour.

## 1. Introduction

Heart failure (HF) is a clinical syndrome characterised by typical symptoms, such as breathlessness, ankle swelling and fatigue, which may be accompanied by signs such as elevated jugular venous pressure, pulmonary crackles and peripheral oedema [1]. It is a growing global health challenge, with a great economic burden for health system [2]. The prevalence is approximately 1–2% of the population in developed countries, and this percentage rises above 10% in people over 70 years of age. Due to its complex and progressive nature it usually results in adverse events, such as high rate of hospital readmission and mortality [2,3,4].

Self-care is considered essential in the management of chronic illnesses. It can be defined as a naturalistic decision-making process of maintaining health through health-promoting practices and managing illness. Self-care in people with chronic illness is more prominent because their management should be seen as a continuum and a priority [5,6].

When patients seek help for the relief of the symptoms caused by their disease, nurses can be the perfect intermediary to motivate patients to engage in self-care. The better that health professionals understand the self-care process, the better they can intervene in people with chronic illnesses, helping them to identify at which stage of self-care they can achieve better results and when [6].

HF is a chronic illness and requires that people integrate practices and recommendations for self-care, in order to maintain the best possible well-being. Effective self-care involves activities and skills that should be learned and used by the individuals, so they can maintain physiologic stability (maintenance), be more able to understand adverse symptoms (symptom perception) and be able to quickly manage them (management) [7].

Patients must monitor their symptoms, adhere to the pharmacological recommendations, a healthy diet, cease tobacco use, limit alcohol consumption and physical activity (PA) or exercise training regimens. By doing so, patients will be able to manage signs and symptoms by recognizing changes and responding either by adapting behaviours or by seeking appropriate assistance. This is a dynamic process, where the patients daily choose to engage in behaviours to achieve illness stability. According to the naturalistic decision-making theory, each unique decision will be made on the basis of past experience and the information available at the present [7,8].

Many instruments have been used over the past years to evaluate self-care behaviour in HF patients. However, only two of them really measured self-care itself, rather than other instruments that only measured constructs that predict or correlate to self-care [9]. The Self-Care Heart Failure Index (SCHFI) is one of those instruments [10]. Some studies demonstrate differences in self-care behaviour between men and women [11,12,13] and, in all of them, the level of self-care is insufficient [14,15].

Gender has been described to influence the level of self-care [14], even though the reasons for this difference are not known [16,17]. Some studies reveal that men and women present different levels of self-care in the different domains of the SCHFI and more surprisingly, these results are sometimes inconsistent. For example, in some studies males frequently present inadequate self-care maintenance while females present inadequate self-confidence [12] and in others it is possible to observe the opposite: being male represents being more adequate in terms of self-care maintenance and being a women less confident [18]. These peculiar results are one of the reasons why gender differences are considered to be a very important factor when trying to understand what causes these inconsistencies.

Regarding pathophysiologic characteristics, New York Heart Association (NYHA) functional class is a classification related to the functional status of the patients [1] and it can vary from Class I to IV regarding the symptoms of the patients. Since HF self-care is closely related to symptoms it is reasonable to expect that more decompensated patients, like NYHA IV, may present worse self-care behaviour scores. Also, the left ventricular systolic ejection fraction (LVEF) is an important clinical characteristic used to classify HF patients, namely: reduced (HFrEF: LVEF < 40%), mid-range (HFmrEF: LVEF 40–49%) and preserved (HFpEF: LVEF ≥ 50%) [1,19].

In Portugal, there is a lack of evidence about self-care behaviour among HF inpatients. Understanding the factors related to the level of self-care behaviour can help nurses to improve nursing care.

The hypotheses of this study are that gender is related with a better self-care behaviour and better pathophysiological characteristics are related with better self-care behaviour. To respond to these hypotheses, the aims of this study are to understand the relationship of gender and pathophysiological characteristics with self-care behaviour and to evaluate the self-care behaviour in a sample of Portuguese heart failure inpatients, using the SCHFI.

The choice of inpatients is due to the possibility to assess not only self-maintenance but also to assess the extent to which patients implement measures to try to compensate for the signs of clinical decompensation that motivated to seek the health team (self-management). If on the other hand stable patients were evaluated it would not be possible to assess what measures they would take in case of decompensation, since they do not experience signs of decompensation and their answers would be inferences about what they would eventually do. These answers could not correspond to what would actually happen if the situation occurred. Therefore, we consider that in order to achieve the defined objectives, the evaluation of inpatients would be more appropriate.

## 2. Materials and Methods

A cross-sectional multicentre study was carried out aiming to evaluate the self-care behaviour of a group of patients admitted due to decompensated heart failure, in eight different hospitals in Portugal. Each centre elected the investigators who would collect the data and all of them received the same orientations for the application of the program, including data collection forms. The principal investigator visited each hospital to present the program, the data collection formulary and to clarify doubts. Regular visits were performed in order to collect the formularies and to monitor the progression of the study.

At admission, the patients were evaluated in terms of socio-demographic and clinical variables such as: (1) self-care behaviour of the disease using the SCHFI; (2) functional capacity according to the London Chest Activities of Daily Living scale (LCADL) and the Barthel index (BI); (3) existence of cardiovascular risk factors (CVRFs) such as hypertension, dyslipidaemia, sedentary lifestyle, obesity, heredity, diabetes, tobacco use and stress; (4) New York Heart Association (NYHA) functional class; (5) left systolic ventricular function; (6) HF aetiology; (7) age; and (8) gender.

The STROBE cross-sectional guidelines from the EQUATOR network, were used in order to best organize the text [20].

### 2.1. Participants

The inclusion criteria for the present study were: (1) diagnosis of decompensated HF; (2) age > 18 years; (3) ability to provide informed consent. Exclusion criteria were: (1) cognitive impairment; (2) inaugural HF (patients with new onset of HF). The sample of patients is composed mostly by male patients (*n* = 153; 68%) with a significant level of functional impairment, shown by the scores of BI and LCADL. The majority of patients are NYHA III (*n* = 176; 78.2%), with the reduced ejection fraction the most prevalent type of ventricular function. Regarding the etiology of the HF, there is no significant difference, even though ischemic and valvular causes are more frequent than the other causes together (namely alcoholic, dilated or atrial fibrillation cause).

### 2.2. Data Collection

In all hospitals, patients received educational sessions about managing heart failure during hospitalisation and phase I cardiac rehabilitation. Rehabilitation nurses and general practitioner nurses performed the educational sessions. Rehabilitation sessions were performed by rehabilitation nurses, after a collaborative evaluation with a cardiologist specialized in heart failure.

### 2.3. Instruments

The main instrument used in this study to evaluate patients’ level of self-care behaviour was the SCHFI. This instrument is divided into three sections related to different domains: maintenance (ten items); management (six items); and confidence (six items). Each item is evaluated using a scale ranging from one to four in which one means ‘never or rarely’, two means ‘sometimes’, three means ‘frequently’ and four means ‘always or daily’. In the section Self-care management, exceptionally, two items can be scored zero, meaning that the patients did not recognise or try any measure. According to the guidelines for the use of the SCHFI, each domain should be converted to a score range from zero to 100, meaning zero is the worst self-care behaviour and 100 the best. The instrument does not return a total score, but a partial score for each section that should be evaluated separately. Scores ≥70 points for each subscale indicate appropriate self-care [10]. The Portuguese version of the SCHFI, used in this study, presents a Cronbach’s alpha coefficient for the total scale of 0.858, revealing a good reliability and validity [9,10].

The Barthel Index (BI) is an instrument that assesses the level of independence of the person in performing ten activities of daily living (ADL): eating; personal hygiene; toilet use; bathing; dressing; and undressing; sphincter control; walking; transferring from the chair to the bed; and climbing the stairs [21]. The minimum score of zero points corresponds to the maximum dependency for all ADL evaluated, and the maximum score of 100 points equals total independence for the same ADL [22,23,24]. Although BI does not directly refer to dyspnea or exertion, it is a good universal measure for the level of dependence and a good predictor of one-year mortality [22].

London Chest Activities of Daily Living scale (LCADL) assesses the limitation that dyspnoea causes in the performance of ADL [19]. It is a questionnaire of 15 items, divided into four domains (self-care, domestic care, leisure and PA), each item was scored from zero to five, with a maximum of 75 points. The higher the value, the greater the limitation in ADL due to dyspnoea [25]. It is possible to obtain a partial score for each domain [26]. In the present study the domain ‘domestic’ was excluded since the patients were hospitalised.

### 2.4. Statistical Analysis

The data were analysed with IBM Statistical Package for Social Sciences^®^ (SPSS) v.21.0. The results of descriptive statistics are presented with mean ± standard deviation. Independent sample T-test, chi-square and Pearson correlations were used. The association of the different sections of the SCHFI score with the variables of interest was performed using one-way analysis of variance ANOVA. A significance level at *p* < 0.05 was assumed.

### 2.5. Ethical Considerations

The study was approved by each ethical commission of every hospital who participated in the study and all patients gave informed consent.

## 3. Results

A total of 225 patients (68% male) were included in this study. Most of these patients had reduced ejection fraction of the left ventricle (*n* = 184, 81.7%) and were admitted in NYHA (New York Heart Association) class III (*n* = 176). The main socio-demographic, clinical and functional characteristics by gender are presented in Table 1.

The results of the three different sections of the SCHFI are presented in Table 2. The calculation of the individual scale score was performed according to the guidelines of the instrument [10].

Considering the cut-off value of ≥70, the total sample of patients present a mean value that ranges from 4.9% to 10.2% of proper self-care behaviour (Table 3).

Table 4 presents the results of the association tests (ANOVA) between different variables and the score of each section of the SCHFI. Only in the NYHA there are variables associated with a better self-care, namely in the management section (*p* = 0.011) and in the self-confidence section (*p* = 0.010).

Correlations were made using the numeric variables age, CVRF, BI, LCADL, self-care maintenance, self-care management and self-care confidence, in order to understand the influence of the variables with each other (Table 5). Self-care maintenance, self-care management and self-care confidence present a positive correlation, at 99% confidence interval between them (self-care maintenance with self-care management: r = 0.365, *p* < 0.000; self-care maintenance with self-care confidence: r = 0.272, *p* < 0.000 and self-care management with self-care confidence: r = 0.670, *p* < 0.000). In addition, self-care maintenance presents a positive correlation with age at a 95% confidence interval (r = 0.158, *p* = 0.018). Negative correlations were found between (1) BI and age (r = −0.151, *p* = 0.023), at a 95% confidence interval and (2) BI with LCALD (r = −0.407, *p* < 0.000), at a 99% confidence interval.

## 4. Discussion

This is the first time that an evaluation of self-care behaviour has been carried out in Portuguese HF patients. Other variables may be of interest to study and this investigation will lead us to seek more embracing criteria to understand the self-care behaviour of Portuguese patients.

### 4.1. Self-Care Behaviour

The mean scores obtained for each section of the SCHFI were below 70 points, meaning that the participants presented a non-appropriate self-care behaviour. These findings are in accordance with other studies [12,14,27,28,29,30,31]. However, the mean values in those studies were higher, ranging from 50 to 65 points, in contrast with the ones from the present study, which ranged between 35 and 45. The self-care confidence is the section with the lowest score in this study, contrary to the ones consulted (self-confidence normally has the best result) [12,31]. This might indicate that participants have more difficulties in understanding the disease and evaluating their symptoms and treatment.

Analysing self-care maintenance, it is clear that patients more easily comply with behaviours that are dependent on health-care providers, such as vaccines, consultations and medication, than with others like weighing, ankle checking for swelling or performing any physical activity that depend only on themselves. However, despite being concerning, these results are similar to other studies [14,27,30]. It is very important to make patients aware of the importance of being active agents in the effective management of their disease. This could be achieved with education and patient involvement in the whole disease management and treatment, as many studies have revealed [32,33]. It is probable that the strategies that are being developed in Portugal need to be improved and patients must be followed up regularly [33].

Regarding self-care management, the score is very similar to other studies already mentioned [14], and the behaviour of patients is similar to self-care maintenance: they present higher values in the items related to the contact with health-care providers than on interventions that could be managed by themselves first, such as reducing fluid intake or controlling salt intake. The majority of patients did not recognize the signs and symptoms of a decompensation, which indicates that more effective strategies are needed. However, it is positive that patients have a high level of confidence in their health-care teams.

A very low percentage of patients (4.9% to 10.2%) were found to have an adequate self-care behaviour (≥70), but this is similar to other European countries [14,27,29].

### 4.2. Gender Differences

Based on our findings, no difference was found between genders on any section of the SCHFI. Among all variables, only the number of CVRFs and the left ventricular ejection fraction had significant differences. The evidence available shows different results, some similar to ours [27]. In the study by Mei et al. [13], where only self-care maintenance was evaluated, there was no statistically significant difference between genders, even though it was slightly better in women, as in the present study. Graven et al. [29], in a study analysing the differences by gender and race in the SCHFI and Dellafiore et al. [12], found no difference in self-care maintenance and management, but a statistically significant difference in self-care confidence was achieved, favouring men. In the present article, males had better results, but not with a statistically significant difference. However, other studies present a significant difference favouring females regarding the score of self-care, with worse outcomes for men [34]. The majority of the studies divided the sample of patients by gender for characterization but did not analyse self-care behaviour separately [12,27]. In the present study, it seems clear that gender does not have significant influence on self-care behaviour in our study.

### 4.3. Correlations

A positive correlation was found between the score of the three sections of the SCHFI, in line with other investigations [31]. Patients who had higher scores in one section present the same pattern in the others, probably because the awareness about the disease and self-care influences mutually all the domains of self-care behaviour. The positive correlation between age and section A may be explained by the fact that the older the patient is, the longer the number of years of disease, leading patients to a better maintenance level.

The negative correlation presented between the BI and age is expected considering that younger patients are normally more independent and have better functional capacity [22,35]. The correlation between BI and LCADL reveals that patients with higher BI present lower LCADL at admission, since low scores of LCADL represents that patients present less exertion on ADL, which is consistent with a greater capacity to perform those activities (evaluated by BI) [19,22].

The association tests (ANOVA) allow the inference to be made that the level of physical activity is directly associated with better self-care maintenance and confidence. These patients are more concerned about their disease, having a more adequate behaviour in terms of self-monitoring, such as ankles checking, weighing, healthy eating habits with reduced salt intake and the ability to understand the effect of the medication, the symptoms and the disease. These patients are probably more motivated to control their disease, to live longer with a better quality of life and, for this reason, they probably present a better autonomous motivation, which is related with positive changes in health behaviour and consequently in self-care behaviour [27,36].

### 4.4. Pathophysiological Characteristics

Regarding NYHA functional class and left ventricular systolic function, only NYHA class II patients present a statistically significant difference in self-management and self-confidence comparing to Class III and IV patients (who do not present differences between them). Class II patients present a worse level of management and confidence, which can be related to the fact that these patients present less symptoms. If patients are less symptomatic, they do not need to take additional measures to achieve compensation, since self-care management section is related to the actions taken by the patients to stabilize symptoms of decompensation. On the other hand, if patients are less symptomatic it would be expected that they would have a higher level of confidence. However, this did not happen. In a study with Brazilian patients similar results were found related to the differences in self-care maintenance [27]. Contrarily to our results, in the study of Graven et al., NYHA class II patients present higher levels of self-care behaviour, in absolute values, even though they were not statistically significant [29].

### 4.5. Implications for Clinical Practice

The results of our study demonstrate that patients, independently of gender, present inadequate levels of self-care behaviour, with self-care management being the most affected area. With these results, nurses and rehabilitation nurses have a guidewire to improve patient education, knowing in which area they must improve education.

Since it is a Portuguese national study, presenting no significant differences between the different hospitals, the results shows that the education strategies must be improved in all centres. Education sessions should not be performed just before discharge but across the entirety of the hospital stay, focusing in different areas of the self-care in different sessions, allowing patients to integrate the knowledge and to have time to ask questions if any doubt occurs. Rehabilitation nurses should focus on questions regarding physical activity and patients must experience some periods of exercising in order to understand the exertion of the different activities of daily living. Caregivers must also be involved in order to learn how to identify signs of decompensation and to understand what health surveillance scans to which they should be aware to help patients to improve their level of self-care. It would also be important to have a checklist showing the areas where patient and caregivers present acquired knowledge and the areas that need to be improved, for example in primary health care centres that should follow-up patients across time. These are some strategies suggested by the authors, regarding the analysis of the results by the different areas of self-care behaviour evaluated by the SCHFI.

Besides the results of our study and the studies discussed, there is no consistent results that may allow to identify patterns about the influence of NYHA class and the level of self-care behaviour. Due to these results, the educational strategies can be the same for patients in different NYHA class.

### 4.6. Limitations of the Study

Some limitations should be addressed with regard to this study. The data were collected from eight different hospitals and by different nurses, which may lead to finding different environments and procedures. The programmes available for patient education by nurses may have been inconsistent between hospitals. Data from the level of education or social support of the patients were not collected. The sample size and proportion of patients from different NYHA class and aetiologies should be more representative.

## 5. Conclusions

Patients present an inadequate self-care behaviour, in general lower than other populations in Europe and worldwide. Structured educational programs must be developed all over the country in order to increase the levels of self-care. These programs should be circular, i.e., start in primary health care, be continued when patients are admitted for hospitalisation due to decompensation and followed up again when patients return to home. In this way there would be a continuity of the educational process and consequently we will be contributing for the empowerment of the patient to improve their self-care. Apparently, gender and pathophysiological characteristics do not interfere in the self-care behaviour of heart failure patients. However, more investigations must be performed to deepen the knowledge in this field, namely, to understand the level of literacy of HF patients, adapting the educational programs to the level of knowledge that patients and caregivers present.

## Figures and Tables

**Table 1:** Comparison of the patients according to gender.

Evaluation		Gender		
	Total (*n* = 225)	Men (*n* = 153) Mean ± SD	Women (*n* = 72) Mean ± SD	*p* Value
Age (years)	68.39 ± 10.67	68.42 ± 10.37	68.32 ± 11.35	0.950
Barthel Index	75.67 ± 19.97	76.54 ± 19.14	73.83 ± 21.65	0.367
LCADL	28.89 ± 9.24	28.14 ± 9.12	30.49 ± 9.38	0.079
CVRF	3.36 ± 1.41	3.50 ± 1.45	3.08 ± 1.28	0.031
Self-care				
Self-care maintenance	47.94 ± 18.12	47.15 ± 19.20	49.63 ± 15.57	0.305
Self-care management	35.56 ± 22.97	36.21 ± 23.50	34.17 ± 21.90	0.525
Self-care confidence	38.78 ± 25.61	39.37 ± 25.68	37.53 ± 25.59	0.615
	N (%)			X^2^
NYHA (New York Heart Association) class				
II	14 (6.2%)	11 (7.2%)	3 (4.2%)	0.426
III	176 (78.2%)	116 (75.8%)	60 (83.3%)	
IV	35 (15.6%)	26 (16.9%)	9 (12.5%)	
LVSF (Left Ventricular Systolic Function)				
HFpEF	27 (12%)	11 (7.2%)	16 (22.2%)	0.003
HFmEF	14 (6.2%)	8 (5.2%)	6 (8.3%)	
HFrEF	184 (81.8%)	134 (87.6%)	50 (69.4%)	
Ethiology				
Ischecmic	73 (32.4%)	60 (39.3%)	13 (18%)	0.513
Valvular	74 (32.9%)	39 (25.4%)	35 (48.7%)	
Other	78 (34.7%)	54 (35.3%)	24 (33.3%)	

CVRF—cardiovascular risk factors; HFmEF—Heart failure with mid-range ejection fraction; HFpEF—Heart failure with preserved ejection fraction; HFrEF—Heart failure with reduced ejection fraction; LCADL—London chest activities of daily living scale; SD—standard deviation.

**Table 2:** Self-care index results.

**Self-Care Maintenance**	**%**					**Mean ± SD**
	**1**		**2**	**3**	**4**	
1. Do you weigh yourself?	49.3		28.4	13.3	8.9	1.8 ± 1.0
2. Do you check if your ankles are swollen?	42.7		17.3	21.3	18.7	2.2 ±1.2
3. Do you try to avoid getting sick (for example: be vaccinated against flu, avoid contact with sick people)?	12.9		11.1	33.8	42.2	3.1 ± 1.0
4. Do you practice any physical activity?	64.9		16.9	12.9	5.3	1.6 ± 0.9
5. Are you assiduous in the consultations with the doctor or nurse?	4		4	19.6	72.4	3.6 ± 0.7
6. Do you ingest a low-salt diet?	19.1		24	31.1	25.8	2.6 ± 1.1
7. Do you exercise for 30 min?	71.1		15.1	8	5.8	1.5 ± 0.9
8. Do you forget or fail to take any of your medicines?	10.7		4.4	15.6	69.3	3.4 ± 1.0
9. Do you request foods with little salt when eating out or visiting someone?	49.3		25.8	15.1	9.8	1.9 ± 1.0
10. Do you use a system (pillbox, reminders) to remind you about your medicines?	36.9		1.8	10.7	50.7	2.8
Total score						46.5 ± 20.9
**Self-Care Management**	**%**					**Mean ± SD**
	**0**	**1**	**2**	**3**	**4**	
11. How quickly did you recognize them as symptoms of heart failure?	38.7	14.2	16	21.3	9.8	1.5 ± 1.4
12. Reduce the salt at your diet		46.7	13.3	24.9	15.1	2.1 ± 1.1
13. Reduce fluid intake		47.1	19.1	25.3	8.4	2.0 ± 1.0
14. Take a further diuretic		68.4	18.7	9.3	3.6	1.5 ± 0.8
15. Contact your doctor or nurse for guidance		10.7	8.4	40.4	40.4	3.1 ± 1.0
16. Think of one of the above features you tried the last time when you had trouble to breath or swollen ankles. Are you sure this feature helped you?	53.8	12	17.3	13.8	3.1	1.0 ± 1.2
Total score						35.1 ± 25.4
**Self-Care Confidence**	**%**					**Mean ± SD**
	**1**		**2**	**3**	**4**	
17. Be free of the heart failure symptoms?	41.3		30.2	27.1	1.3	1.9 ± 0.9
18. Follow the recommended treatment?	24.9		13.8	45.3	16	2.5 ± 1.0
19. Evaluate the importance of your symptoms?	27.6		26.7	40	5.8	2.2 ± 0.9
20. Recognize changes in health, if they occur?	24		26.7	44	5.3	2.3 ± 0.9
21. Do something that can relieve your symptoms?	30.7		28.4	36.4	4.4	2.1 ± 0.9
22. Assess whether a drug works?	42.7		29.3	26.2	1.8	1.9 ± 0.9
Total score						38.5 ± 26.0

**Table 3:** Frequencies of appropriate self-care behaviour.

Score	Self-Care Maintenance	Self-Care Management	Self-Care Confidence
	*N* (%)	*N* (%)	*N* (%)
≥70	23 (10.2%)	25 (11.1%)	11 (4.9%)
<70	198 (89.8%)	200 (88.9%)	214 (95.1)

**Table 4:** Association tests between different variables and the score of each section of the Self-Care Heart Failure Index (SCHFI).

		Self-Care Maintenance	Self-Care Management	Self-Care Confidence
**NYHA (New York Heart Association) class**				
II	*N*	14	14	14
	Mean	46.14	53.21	58.86
	SD	17.50	16.60	12.89
III	*N*	176	176	176
	Mean	48.30	34.29	37.35
	SD	18.56	22.99	26.19
IV	*N*	35	35	35
	Mean	46.86	34.86	37.77
	SD	16.40	22.67	23.75
	*p* value	0.848	0.011	0.010
**LVEF (Left Ventricular Systolic Function)**				
HFpEF	*N*	27	27	27
	Mean	47.89	35.74	41.81
	SD	15.03	22.09	27.46
HFmEF	*N*	14	14	14
	Mean	50.86	43.93	47.71
	SD	13.42	20.11	25.32
HFrEF	*N*	184	184	184
	Mean	47.73	34.89	37.66
	SD	18.88	23.28	25.33
	*p* value	0.825	0.367	0.297

HFmEF—Heart failure with mid-range ejection fraction; HFpEF—Heart failure with preserved ejection fraction; HFrEF—Heart failure with reduced ejection fraction; SD—standard deviation.

**Table 5:** Pearson Correlations test (r) between numeric variables (N = 225).

Evaluation	Self-Care Maintenance	Self-Care Management	Self-Care Confidence	Age	Barthel Index (BI)	LCADL
Self-care management	0.365 **	-	-	-	-	-
Self-care confidence	0.272 **	0.670 **	-	-	-	-
Age	0.158 *	0.001	−0.091	-	-	-
Barthel Index	0.063	0.125	0.213 **	−0.151 *	-	-
LCADL	0.077	−0.226 **	−0.376 **	0.072	−0.407 **	-
CVRF	−0.095	−0.098	−0.12	0.101	−0.125	0.022

* Correlation is significant at the 0.05 level (2-tailed); ** Correlation is significant at the 0.01 level (2-tailed).

## Data Availability

The data of this study will be available from the corresponding author upon request.
